# Supplementary material for: Epistasis-driven identification of SLC25A51 as a regulator of human mitochondrial NAD import
Source: Nat Commun. 2020 Dec 1;11:6145. doi: 10.1038/s41467-020-19871-x (PMC7708531; doi:10.1038/s41467-020-19871-x)
Supplement: Supplementary file 1 — Supplementary Information [file 41467_2020_19871_MOESM1_ESM.pdf]

Suppl Figure 1

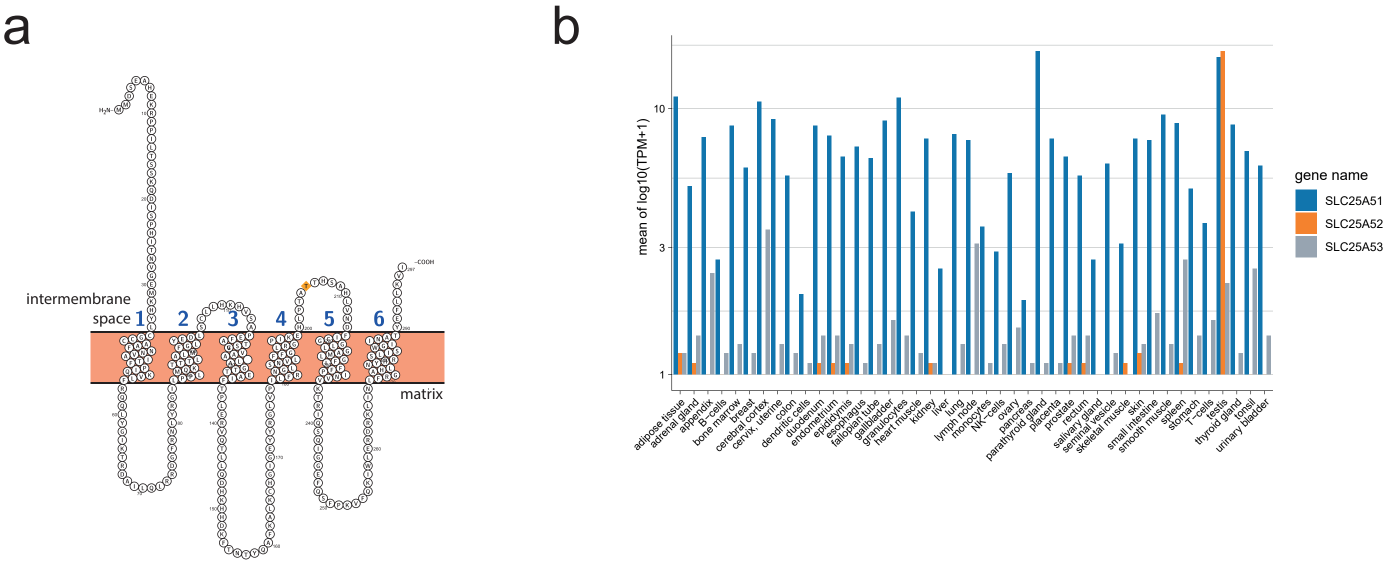

Suppl Fig 1. a. Predicted topology of SLC25A51. Figure generated using Protter[1]. **b.** Expression of SLC25A51/52/53 in healthy tissues according to Uhlen et al[2]. Mean expression is shown (n=1).

References:

1. Omasits, U., Ahrens, C. H., Müller, S. & Wollscheid, B. Protter: interactive protein feature visualization and integration with experimental proteomic data. Bioinformatics 30, 884–886 (2014).

2. Uhlén, M. et al. Tissue-based map of the human proteome. Science 347, (2015).

Suppl Figure 2

a

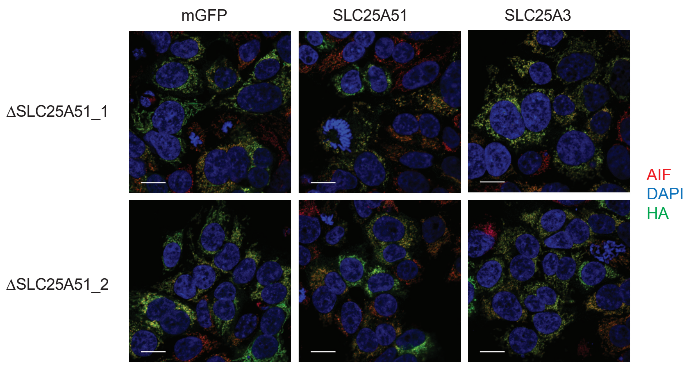

b

Gene	Protein	Cofactor(s)
CS	citrate synthase	
COX20	Subunit of complex IV	
PDHA1	pyruvate dehydrogenase	thiamine/NAD/CoA
PDHB	pyruvate dehydrogenase	thiamine/NAD/CoA
MDH2	malate dehydrogenase	NAD
GRHPR	hydroxypyruvatereductase	NAD
DLAT	pyruvate dehydrogenase	thiamine/NAD/CoA
SLC24A2	Ca2+/K+/Na+ transporter	
GTPBP3	mitochondrial tRNA modification	THF
ATP1B4	Na+/K+ ATPase	

c

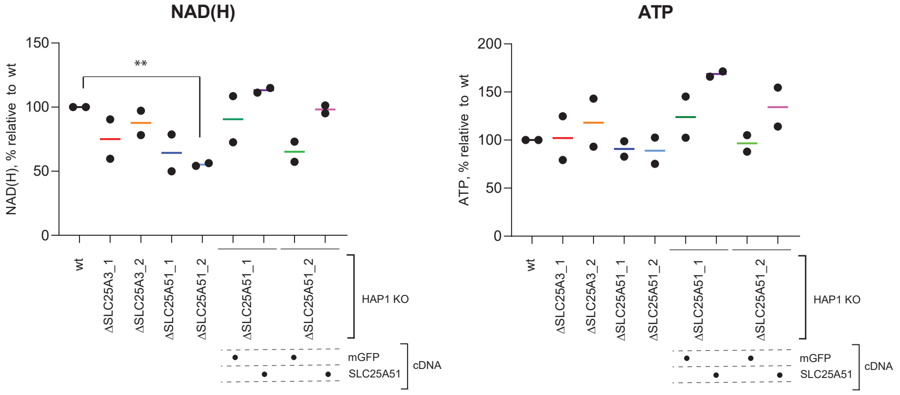

d

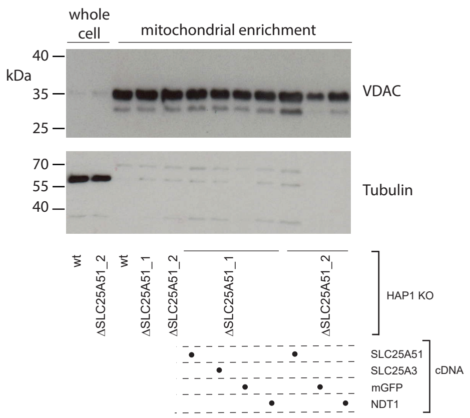

e

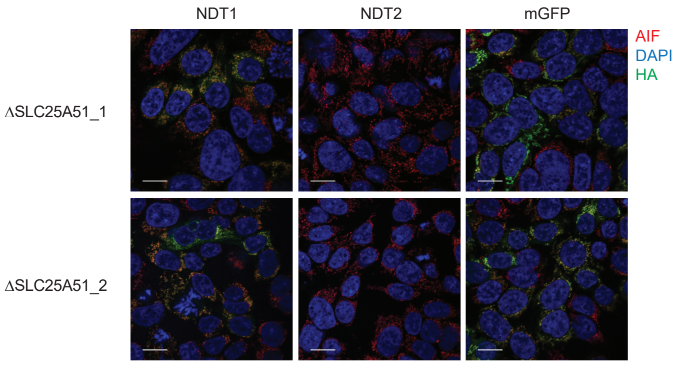

Suppl Fig 2. a. Representative confocal images (out of two independent experiments) of HAP1 cells lacking endogenous SLC25A51 and reconstituted with mitoGFP, SLC25A3 or SLC25A51 cDNA. Green: ectopic HA-tagged protein, Blue: DAPI, Red: Apoptosis inducing factor (AIF). Scale bar: 20 µm. **b.** List of the top correlated genes specific to SLC25A51 but not SLC25A3, according to the co-essentiality analysis. **c.** Total cellular levels of NAD/NADH (lefthand panel) and ATP (righthand panel) in the indicated cell lines, normalized to wt levels. Cumulative results of two biological replicates, each performed in technical triplicates. Statistical significance was calculated with a one-way ANOVA with Dunnett's multiple comparison test. **: pvalue < 0.05. Only statistically significant comparisons are labeled. **d.** Western blot of whole lysate and mitochondria-enriched fractions for the genotype listed, showing differential accumulation of the mitochondrial protein VDAC (Voltage-dependent anion channel) and the cytosolic protein alpha-tubulin. Images from two different membranes derived from one biological replicate experiment. **e.** Representative confocal images (out of two independent experiments) of HAP1 cells lacking endogenous SLC25A51 and reconstituted with HA-tagged mitoGFP, NDT1 or NDT2 cDNA. Green: ectopic HA-tagged protein, Blue: DAPI, Red: AIF. Scale bar: 20 µm.
